# An Ensemble Classifiers for Improved Prediction of Native–Non-Native Protein–Protein Interaction

**DOI:** 10.3390/ijms25115957

**Published:** 2024-05-29

**Authors:** Nor Kumalasari Caecar Pratiwi, Hilal Tayara, Kil To Chong

**Affiliations:** 1 Department of Electronics and Information Engineering, Jeonbuk National University, Jeonju 54896, Republic of Korea; caecarnkcp@jbnu.ac.kr; 2 Department of Electrical Engineering, Telkom University, Bandung 40257, West Java, Indonesia; 3 School of International Engineering and Science, Jeonbuk National University, Jeonju 54896, Republic of Korea; 4 Advances Electronics and Information Research Centre, Jeonbuk National University, Jeonju 54896, Republic of Korea

**Keywords:** protein–protein interaction, machine learning, ensemble classifiers, drug discovery, computational biology

## Abstract

In this study, we present an innovative approach to improve the prediction of protein–protein interactions (PPIs) through the utilization of an ensemble classifier, specifically focusing on distinguishing between native and non-native interactions. Leveraging the strengths of various base models, including random forest, gradient boosting, extreme gradient boosting, and light gradient boosting, our ensemble classifier integrates these diverse predictions using a logistic regression meta-classifier. Our model was evaluated using a comprehensive dataset generated from molecular dynamics simulations. While the gains in AUC and other metrics might seem modest, they contribute to a model that is more robust, consistent, and adaptable. To assess the effectiveness of various approaches, we compared the performance of logistic regression to four baseline models. Our results indicate that logistic regression consistently underperforms across all evaluated metrics. This suggests that it may not be well-suited to capture the complex relationships within this dataset. Tree-based models, on the other hand, appear to be more effective for problems involving molecular dynamics simulations. Extreme gradient boosting (XGBoost) and light gradient boosting (LightGBM) are optimized for performance and speed, handling datasets effectively and incorporating regularizations to avoid over-fitting. Our findings indicate that the ensemble method enhances the predictive capability of PPIs, offering a promising tool for computational biology and drug discovery by accurately identifying potential interaction sites and facilitating the understanding of complex protein functions within biological systems.

## 1. Introduction

Constructed from distinct amino acid sequences, proteins are crucial for an extensive range of biological activities and regulate a multitude of biological tasks, such as development, metabolic processes [1,2], apoptotic autophagy [3], and cell fate [4]. Determining the binding partners is a pragmatic approach to predicting protein function, and protein–protein interactions mediate a significant number of protein tasks [5,6]. Protein–protein interactions (PPIs) are crucial for comprehending the integrated functioning of proteins within a cell to carry out its tasks, implicated in the formation of diverse cellular pathways, function of individual proteins, and the progression of diseases [7,8]. Causes or indicators of a disease state, including infectious diseases, neurodegenerative disorders, and cancer [9], may arise from perturbations in the typical patterns of protein–protein interactions (PPIs) and protein complexes [10]. As a result, addressing PPIs is a vital path for the research and development of novel drugs and a direction in the treatment of diseases. Since technological advancements in structural biology have facilitated the investigation of PPIs through single-crystal X-ray diffraction [11,12,13], Nuclear Magnetic Resonance (NMR) [14,15,16], and Cryo-Electron Microscopy (Cryo-EM) [17,18,19], the experimental scope stays limited and impracticable. High-throughput technologies have generated vast amounts of PPI data across various organisms; the experimental processes for detecting these interactions remain costly and time-consuming [20,21,22]. Subsequently, various computerized PPI prediction methods have been devised to assist and direct the empirical endeavors of wet laboratories. It is accelerated in particular by the growth and further advancement of artificial intelligence predictive algorithms, for use in the fields of biological science, including computational PPI prediction.

The prediction of PPI sites requires a high-accuracy algorithms that identify biological information concealed in protein interactions; thus, the selection of classifiers is essential. Random forests (RFs), Neural Networks (NNs), Logistics Regression (LR), naïve Bayes (NB), and Support Vector Machines (SVMs) are frequently used as single classification algorithms for PPI prediction. Xue-Wen Chen and Mei Liu present a domain-based RF methodology for deducing protein interactions on Saccharomyces cerevisiae; the results provide empirical proof on achieving better specificity and sensitivity in predicting PPIs [23]. By determining the degree of similarity between protein pairs, Yanjun Qi et al. classified pairs of proteins as interacting or non-interacting using RF [24]. Yeast data testing revealed that the approach can increase the coverage of interacting pairs with a 50% false positive rate. The random forest model applied in study [25] for predicting protein–protein interaction sites demonstrated an overall accuracy of 67%. Utilizing the protein sequence datasets and RF classifiers to forecast PPIs, study [26] produced satisfactory results with average accuracies greater than 85% throughout three distinct datasets. Study [27] presents a machine learning approach to single out correct 3D docking models of protein–protein complexes; the results show that the random forest algorithm outperformed and was selected for further optimization. By leveraging the structure and sequence features of proteins, Jha et al. forecasted the interaction between proteins using graph convolutional networks (GCNs) and graph attention networks (GATs) [28]. The findings provide documentation of the proposed method’s efficacy, surpassing the performance of the preceding prominent approaches. The utilization of self-attention Deep Neural Network (DNN) in study [29] was employed to propose a PPI prediction method, and attained favorable outcomes when tested on both interspecific and intraspecific datasets, as well as in cross-species predictions. A protein interaction strategy was created through a deep Convolutional Neural Network (CNN) in work [30]. By minimizing the complexity of computation, this approach enables the extraction of sequence properties that are exceptionally informative. The logistic regression strategy proposed by Qingshan Ni et al. for predicting the function of proteins from protein–protein interaction data generated favorable outcomes and exceeded some previous established models [31]. Study [32] presents a multi-level model PPI with the objective of enhancing the speed and accuracy of predicting large-scale PPIs. This model achieved a remarkable accuracy of 0.99, which bodes well for the efficiency and accuracy of large-scale PPI prediction. The logistic regression model employed in report [33] for projecting protein–protein interactions yielded a precision of 57–77%, a recall of 64–75%, and a specificity of 96–98%. Report [34] presented an NB classifier for protein–protein interactions, which yielded a relatively high level of accuracy. In Ref. [35], sequential data were employed to train a naïve Bayes classifier (NBC). The final performance of the NBC was as follows: Matthew’s correlation coefficient (MCC): 0.151; F-measure: 35.3%; precision: 30.6%; recall: 41.6%. The method under consideration empowers experimental biologists to discern potential interface residues in unidentified proteins solely based on sequence data. Osamu Maruyama, employing genomic datasets, derived a number of features that delineate heterodimeric protein complexes [36]. To determine the parameters of these features, he proposed the naïve Bayes classifier. To predict interaction sites in protein–protein complexes, Geng et al. fed the naïve Bayes classifier (NBC) a feature vector consisting of 181 dimensions of protein sequences [37]. Uddin and Ahmed modified the naïve Bayes classifier by a radial-basis function kernel for the prediction of PPI sites, resulting in a sensitivity of 86%, specificity of 81%, accuracy of 83%, and MCC of 0.65 [38]. In study [39], an SVM algorithm was developed in conjunction alongside surface patch mapping to forecast protein–protein binding sites. The model achieved 76% accuracy in predicting the location of the binding site across the entire dataset. Study [40] improved performance on the interaction of HIV proteins with humans using a sequence of an amino acid dataset by combining SVM and Global Encoding (GE). The findings demonstrate that the suggested approach is resilient, practical, and capable of discerning protein–protein interactions with a maximum accuracy of 85%. The investigation [41] utilized a comprehensive analysis to distinguish between native and non-native protein complexes using a classification scheme based on SVM. Benchmarking and comparative analyses indicate that the classifiers exhibit exceptional performance.

Given the diverse range of classifiers utilized in predicting PPIs, it becomes apparent that each classifier offers unique advantages and insights into the complex task of identifying these interactions. However, to further enhance predictive performance, researchers have increasingly turned to ensemble methods, particularly stacking classifier techniques. By leveraging the strengths of various individual classifiers, ensembles can effectively mitigate the weaknesses inherent in any single classifier approach [42,43,44]. This approach aligns with the inherent complexity of PPI prediction, where different classifiers may capture different aspects of the underlying biological mechanisms. Furthermore, ensemble learning model classifiers are often utilized to boost the performance of target predictions [45,46,47,48]. Similarly, in the realm of Quantitative Structure–Activity Relationships (QSAR), consensus [49,50] approaches play a crucial role in enhancing predictive reliability and accuracy by integrating diverse models and data sources. Both approaches aim to improve prediction accuracy through integration. The key difference lies in their typical application contexts and the nature of their integration strategies.

Native vs. non-native protein–protein interaction prediction based on assembly classification is proposed in this paper as an effective method to enhance the performance of PPI prediction. The system initiates with the preparation of the dataset, which consists of normalizing and splitting the data into sets for training and testing. The pre-processed data will be analyzed by the level 0 predictor algorithm (base learner), which utilizes the random forest, gradient boosting, extreme gradient boosting (XGBoost), and light gradient boosting (LGB) algorithms. The output predictions generated by the base learner are compiled into a matrix that serves as the meta-learner’s input feature (level 1 predictor algorithm). In this study, the logistic regression (LR) classifier functions as a level 1 predictor. In order to evaluate the reliability of the system’s performance, in addition to the analysis of training and test data, a validation procedure was incorporated wherein the optimal model was executed on an independent dataset. The results demonstrate that the model substantially improves the accuracy of protein–protein interaction (PPI) predictions by leveraging the collective strengths of various foundational models, thereby enhancing overall performance. Additionally, the incorporation of a meta-learner enables the model to accommodate complex patterns that may elude explanation by any single baseline model. The proposed model consistently outperforms the previous model across all metrics (Accuracy, Precision, Recall, F1-Score, ROC, AUC) for each trajectory stretch. Notably, the proposed model achieves particularly high scores in the AUC metric, indicating its strong capability to discriminate between classes. The proposed model exhibits stability in its performance metrics, especially in the longer trajectory stretches (60–80 ns and 80–100 ns), with very little variation in the scores, highlighting its robustness. To maintain the independence and objectivity of our assessment, the independent set was exclusively used during the final validation phase only. This evidence suggests that the model is capable of effectively discriminating between native and non-native protein–protein interactions. Consequently, this method holds promise for advancing the precision in identifying PPI sites.

### 1.1. Stacking Ensemble Classifier

Implemented by deducing the generalizers’ biases with respect to a given learning set, stacked generalization is a method for reducing the generalization error rate of one or more generalizers [51]. Stacked ensemble learning involves two separate phases: training the base models and, subsequently, training the meta-models [52,53]. The basic principle entails training the meta-model using the base model’s prediction result as a new feature matrix, and finalizing the prediction outcome through the integration of the approaches in the output of the meta-model [54,55]. The process of building a ensemble model involves three main stages: ensemble setup, training, and prediction [56]. During the ensemble setup, both baseline classifiers and a meta-learner are chosen. In the training phase, each baseline method is trained using appropriate hyper-parameters, followed by training the meta-learner. The meta-learner learns from the predictions and input vectors of the baseline methods and the actual labels of the observations. In the prediction phase, the stacked ensemble is utilized to predict outcomes on new data. Algorithm S1 in the Supplementary Document provides a summary of ensemble classifiers’ algorithm for native–non-native PPIs. The algorithm presented describes an ensemble classifier for binary classification. The value of *j* is an index used to iterate over the base classifiers; it runs from 1 to *J*, which suggests that there are total of *J* base classifiers, while *k* is another index and it is used in the creation of the new dataset $\hat{D}$ for the second step of the ensemble method. In Step 2, a new dataset $\hat{D}$ is created where, for each instance *i*, the feature vector $x_{i}$ is transformed by the predictions of each base classifier, resulting in a new feature vector ${\hat{x}}_{i}$. This is done for each instance from 1 to *K*, where *K* is the total number of instances in the dataset. Constructed by modeling the utilized posterior class probability values as the result of a sigmoid function with the weighted features as an input, the second-level classifier employed for this study is a logistic regression classifier. We investigated the binary classification $y=\left\{0,1\right\}$, with data features $D=\left\{x_{1},x_{2},...,x_{k}\right\}$, and the corresponding weight of $W=\left\{w_{1},w_{2},...,w_{n}\right\}$. The posterior probability of a∈A, supplied with the attribute features of *D*, is able to be expressed through the utilization of the subsequent mathematics equation [57]:

(1) $$P\left(a|D\right)=\lambda {\left(D\right)}^{a}{\left(1-\lambda \left(D\right)\right)}^{1-a}$$

with $\lambda \left(D\right)=\frac{1}{1+e^{-{\vec{w}}^{T}\vec{x}}}$ if the logistic regression is fitted with the training data $D_{t}=\left(\left(\vec{x_{1}},a_{i}\right),...,\left(\vec{x_{n}},a_{n}\right)\right)$ and weight *W* that maximize

(2) $$P\left(D_{t}|w\right)=\prod_{i=1}^{n}\lambda {\left(\vec{x_{i}}\right)}^{c_{i}}{\left(1-\lambda \left(\vec{x_{i}}\right)\right)}^{1-c_{i}}$$

are selected in the process of employing the estimation of maximum likelihood. Following this, predictions are generated by determining the posterior class with the greatest probability:

(3) $$\hat{y}=\underset{a\in A}{arg\max}\;P\left(a|D\right)$$

**Figure 1:**
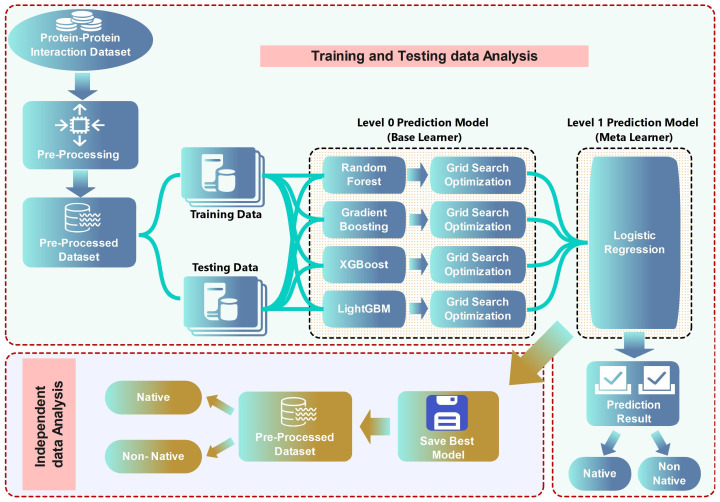
Schematic representation of a two-tiered machine learning framework for classifying protein–protein interactions as native or non-native. The training data are used to build and optimize several base learners, including random forest, gradient boosting, XGBoost, and LightGBM, through grid search optimization. A meta-learner, Logistic Regression, takes these models’ predictions to generate the final classification results.

Figure 1 depicts a structured process for predictive modeling using a stacked ensemble learning approach in the context of protein–protein interaction datasets. Initially, the dataset undergoes pre-processing to refine the input data, which are then divided into training and testing sets. In the first stage of ensemble learning, base models such as random forest, gradient boosting, XGBoost, and LightGBM are trained on the dataset, and their performance is enhanced through grid search optimization. The predictions from these base models are then fed into a second-stage meta-learner, specifically Logistic Regression, which synthesizes the input to generate a final prediction outcome, classifying results as either native or non-native. The best-performing model from this two-tiered process is retained, and its robustness is further validated through independent data analysis, ensuring the model’s efficacy in accurately predicting protein–protein interactions.

### 1.2. Model Performance Evaluation

In binary classification, models are commonly evaluated using metrics derived from a confusion matrix which summarizes model predictions compared to actual labels [58]. A detailed representation of the confusion matrix for the binary classification task is provided in Table S1 of the Supplementary Document. The matrix includes true positives (*TPs*), true negatives (*TNs*), false positives (*FPs*), and false negatives (*FNs*). These terms are used to calculate various evaluation metrics, including—Accuracy (*Acc*), Precision (*Pre*), Recall (*Rec*), *F1-Score*, and *MCC*. Accuracy measures the proportion of correct predictions out of all predictions [59,60]. Precision quantifies the proportion of true positive predictions out of all positive predictions [61,62]. Recall (also known as sensitivity) indicates the proportion of true positive predictions out of all actual positive instances [63]. The F1-score is the harmonic mean of precision and recall, providing a balanced measure [64,65].

(4) $$Acc=\frac{TP+TN}{TP+TN+FP+FN}$$

(5) $$Pre=\frac{TP}{TP+FP}$$

(6) $$Rec=\frac{TP}{TP+FN}$$

(7) $$F1\text{-}Score=\frac{2\times [Pre\times Rec]}{Pre+Rec}$$

Matthew’s correlation coefficient (MCC), considered as sophisticated measure for evaluating the quality of classifier [66,67], ranges from −1 to 1; the value of 1 indicates perfect prediction, 0 indicates not better than random prediction, and −1 indicates total disagreement between prediction and observation.

(8) $$MCC=\frac{\left(TP\times TN)-(FP\times FN\right)}{\sqrt{\left(TP+FP)(TP+FN)(TN+FP)(TN+FN\right)}}$$

The balanced (uniformed) dataset distribution ensures that no single class dominates the dataset, providing a fair basis for evaluating the performance of our models. As a result of this balanced class distribution, the random accuracy, essentially the success rate of a classifier that makes predictions based solely on class frequency, was calculated at 50% [68].

## 2. Result and Discussion

### 2.1. Baseline Model Performance

**Figure 2:**
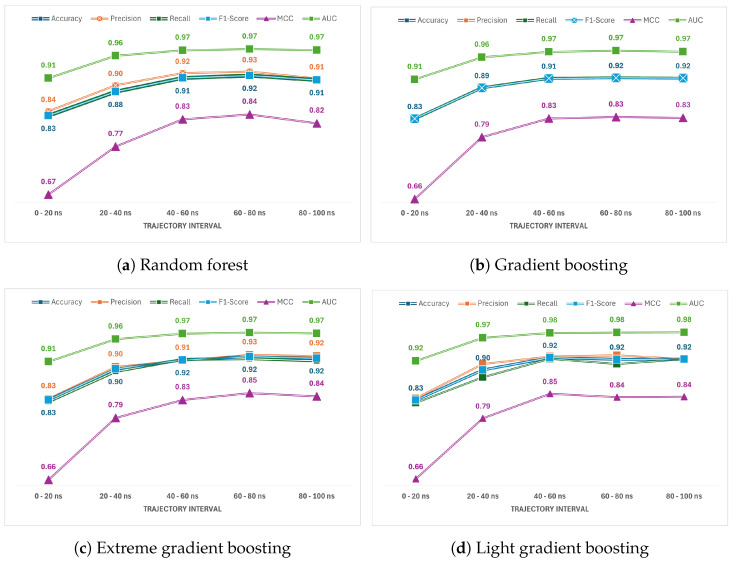
Comparative performance of machine learning models for protein–protein interaction prediction across different trajectory intervals.

The presented data showcase the performance metrics of various machine learning models, random forest, gradient boosting, extreme gradient boosting, and light gradient boosting—applied to predict native versus non-native protein–protein interactions across different trajectory intervals (0–20 ns, 20–40 ns, 40–60 ns, 60–80 ns, and 80–100 ns)—and are shown in Figure 2. The values for *TPs*, *TNs*, *FNs*, and *FPs* for each trajectory interval in each model are presented in Figures S1–S5 of the Supplementary Document. Overall, it is evident that all models perform remarkably well across the various trajectory intervals, with consistently high accuracy, precision, recall, F1-score, and AUC values. Across the trajectory intervals, extreme gradient boosting and light gradient boosting consistently demonstrate the highest accuracy, precision, recall, and F1-score, while also exhibiting superior AUC values, particularly in later trajectory intervals. The high accuracy scores indicate that the models are effectively distinguishing between native and non-native protein–protein interactions. Precision scores, which reflect the proportion of correctly identified positive cases out of all cases classified as positive, demonstrate the models’ ability to limit false positives. Similarly, recall scores measuring the proportion of actual positive cases correctly identified, illustrate the models’ capacity to capture true positives. The F1-score, which considers both precision and recall, provides a harmonic mean that balances these two metrics, showcasing the models’ overall predictive performance. Our results demonstrate that the predictive performance, as measured by the F1-score, reaches a plateau after 40–60 ns of molecular dynamics simulations, suggesting that extended simulation durations do not substantially improve prediction accuracy for protein–protein interactions. Therefore, we assert that shorter simulations are adequate for optimal predictive outcomes, enhancing the efficiency of protein–protein interaction studies.

Moreover, the AUC values, representing the area under the receiver operating characteristic (ROC) curve, further confirm the models’ robustness in distinguishing between native and non-native interactions, with higher AUC values indicating better overall performance. Interestingly, light gradient boosting consistently demonstrates competitive performance, particularly evident in later trajectory intervals (60–80 ns and 80–100 ns), where it surpasses the other models in terms of precision, recall, and AUC. This suggests that light gradient boosting may excel in capturing the subtleties and complexities of protein–protein interactions, especially as trajectories progress. The robustness of extreme gradient boosting and light gradient boosting in discerning native from non-native protein–protein interactions stems from several key factors inherent in their algorithms. Built on the gradient boosting framework, both models leverage sequential training of weak learners to correct errors, thereby effectively capturing intricate relationships within the data. Regularization techniques such as shrinkage and feature subsampling prevent over-fitting and enhance generalization performance. Additionally, their optimized implementations, including parallel processing and histogram-based splitting, ensure efficiency and scalability on large datasets. Furthermore, built-in mechanisms for handling missing values and tunable hyper-parameters contribute to their adaptability and robustness in this study’s dataset, suggesting potential applicability across diverse datasets and tasks under similar conditions. Overall, the combination of these features equips extreme gradient boosting and light gradient boosting with the capability to effectively discern complex biological interactions, making them valuable tools for predictive modeling in computational biology.

Interestingly, a discernible upward trend in accuracy was observed with the temporal progression of trajectory intervals, particularly evident in the latter stages (60–80 ns and 80–100 ns), implying the potential accrual of informative data over time. The trajectory intervals, as utilized in molecular dynamics simulations, serve as discrete time segments within which the system’s molecular configurations and dynamics are analyzed. These intervals are typically defined based on the duration of the simulation and are crucial for capturing temporal changes in the system. In this study, the trajectory intervals likely represent consecutive 20 nanosecond segments of the molecular dynamics simulation. Each interval corresponds to a distinct temporal snapshot of the system’s behaviour. When the trajectory time of an MD simulation is increased, this generally allows for more thorough sampling of the conformational space of the protein complex. Longer simulations give the protein complex sufficient time to reach equilibrium. Initial phases of MD simulations can involve relaxation from potentially artificial or strained starting conditions, which may not represent the true energy minimum. In our analysis, as depicted in Figure 2, we observe that the incremental benefits of extending the trajectory intervals beyond 60–80 ns are minimal across the tested models. Initially, we hypothesized that longer simulations would consistently enhance the model’s performance metrics. However, the results indicate a plateau in improvements for accuracy, precision, recall, F1-score, MCC, and AUC in longer intervals. This finding emphasizes the efficiency of these models within relatively shorter simulation durations, which is beneficial for practical applications where computational resources are limited.

To assess the effectiveness of four baseline models, we juxtaposed their performance with the logistic regression algorithm, as detailed in Figure S6 of the Supplementary Document. This comparison used uniform performance metrics, revealing that logistic regression falls short across all metrics when contrasted with the baseline models. Specifically, the highest accuracy and F1-score achieved by logistic regression hover around 0.70 in the optimal 80–100 ns interval, markedly inferior to even the weakest results of the baseline models in their best scenarios. This lower performance suggests that logistic regression may not adequately capture the complex relationships inherent in the dataset, likely due to the presence of interactions and non-linearities better addressed by tree-based models. Therefore, while logistic regression serves as a valuable reference, the baseline models prove considerably more adept at managing the data complexities typical of molecular dynamics simulations.

The analysis underscores the effectiveness of tree-based learning techniques, particularly gradient boosting methods, in accurately predicting native versus non-native protein–protein interactions across different trajectory intervals. The consistently high performance across metrics and trajectory intervals suggests the potential practical applicability of these models in computational biology and drug discovery efforts, aiding in the understanding of protein–protein interaction dynamics and facilitating the design of novel therapeutics. To support the performance depicted in Figure 2, we have included Figures S8–S12 in the Supplementary Document. These figures provide a comparative analysis of the performance of different base models, including the ensemble model, for predicting Native vs. Non-Native PPIs at each trajectory interval. The metrics for both training and testing datasets are displayed.

### 2.2. Ensemble Classifier Performances

**Figure 3:**
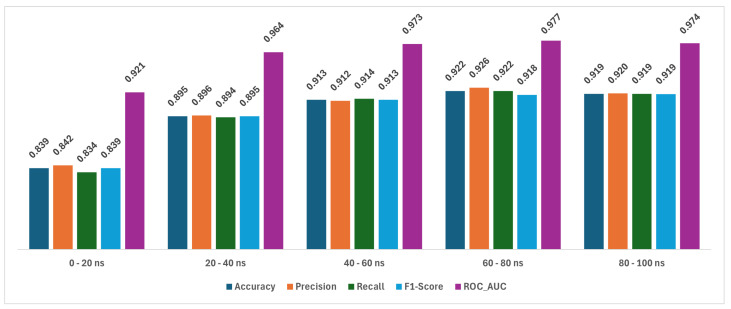
The performance ensemble classifier for each trajectory interval.

The implemented ensemble classifier amalgamates predictions from random forest, gradient boosting, extreme gradient boosting, and light gradient boosting models, leveraging the strengths of each to enhance predictive accuracy regarding native versus non-native protein–protein interactions. By utilizing a logistic regression meta-classifier, the ensemble classifier optimally combines the diverse predictions from the base estimators. Evaluation of its performance on both the training and test sets reveals its effectiveness in generalizing to unseen data while maintaining robustness. The results from the ensemble classifier showcase consistently high performance metrics across all trajectory intervals, its presented in Figure 3, indicating its robustness in predicting native versus non-native protein–protein interactions.

Firstly, the accuracy scores consistently exceed 0.839 across all trajectory intervals, indicating that the ensemble classifier correctly classifies protein–protein interactions with a high degree of precision. This suggests that the model effectively distinguishes between native and non-native interactions, crucial for applications in drug discovery and computational biology. Similarly, precision scores consistently surpass 0.84, illustrating the ensemble classifier’s ability to limit false positives, ensuring that the majority of predicted positive interactions are indeed true positives. This precision is particularly noteworthy in the later trajectory intervals (60–80 ns and 80–100 ns), indicating the model’s capability to maintain high precision as trajectories progress. The recall scores, consistently above 0.83, demonstrate the ensemble classifier’s capacity to capture a substantial portion of true positive interactions. This indicates that the model effectively identifies most native interactions within the dataset, crucial for comprehensive analysis in biological systems. Furthermore, the F1-scores, which consider both precision and recall, consistently hover around 0.84, reflecting a harmonious balance between precision and recall across trajectory intervals. This balanced performance underscores the ensemble classifier’s effectiveness in capturing both positive and negative instances accurately. Lastly, the ROC_AUC values, consistently above 0.92 and peaking at 0.98 in the 60–80 ns trajectory interval, highlight the model’s robustness in distinguishing between native and non-native interactions across different time points. This indicates that the ensemble classifier’s predictions exhibit strong discriminatory power, crucial for tasks involving imbalanced datasets or when the cost of misclassification varies. In summary, the ensemble classifier demonstrates consistently high performance across various evaluation metrics and trajectory intervals, showcasing its efficacy in accurately predicting protein–protein interactions. These results underscore the model’s potential utility in advancing our understanding of biological systems and aiding in drug discovery efforts.

By time intervals, in molecular dynamics simulations, trajectories refer to the paths that particles, such as atoms or molecules, take over time as they move within a simulated environment. These trajectories are influenced by the physical interactions and forces at play within the system. In the earliest interval (0–20 ns), the metrics are the lowest but still quite high, which could indicate that it is more challenging to distinguish between native and non-native interactions at the beginning of the trajectories. As we move to the 20–40 ns interval, there is a noticeable improvement in all metrics, with precision showing the highest value (0.921), which could suggest that as the interaction progresses, the model becomes better at limiting false positives. The 40–60 ns interval shows a peak in ROC_AUC, suggesting that the classifier is most effective at distinguishing between the two classes during this phase. In the 60–80 ns interval, there is the highest accuracy noted, which may indicate that the protein–protein interactions are most distinguishable at this stage, and the model can classify them with great confidence. The final interval (80–100 ns) shows slightly decreased accuracy and precision compared to the 60–80 ns interval but maintains a high ROC_AUC, suggesting a consistent ability to distinguish between interactions. The fact that the performance metrics generally improve or maintain high levels across trajectory intervals indicates that the ensemble classifier is effectively learning from the temporal dynamics captured in the molecular simulations.

Overall, the performance of the ensemble classifier is robust across all measured intervals and metrics. This indicates that the ensemble approach, leveraging multiple models and using a logistic regression meta-classifier, is effective for this task. It is clear that the classifier is well-calibrated and generalizes well to unseen data, maintaining both a high level of accuracy and a balance between precision and recall, which are critical in computational biology and drug discovery applications. PPI prediction models can be a valuable tool for screening protein pairs while developing new drugs for targeted protein degradation [69].

### 2.3. Comparative Performance with Existing Methods

From the provided Table 1 comparing the performance of the random forest model and the proposed ensemble model on different trajectory stretches and the independent set, we can draw several observations. The independent dataset was rigorously reserved for use solely during the final validation phase to preserve the objectivity and impartiality of our evaluation process. This dataset was not utilized during the initial training or parameter tuning phases of the models. By segregating it for exclusive use in the final assessment, we ensured that the evaluation metrics genuinely reflect the model’s performance on previously unseen data, thus providing a robust and scientifically valid measure of its predictive accuracy and generalization capabilities. For the validation process, the model was simulated for the full 100 ns. This approach aimed to assess the model’s performance over an extended period. The proposed ensemble model consistently outperforms the random forest model in terms of accuracy across all trajectory stretches as well as on the independent set. This indicates that the ensemble model is better at correctly classifying instances. Both models show similar precision scores, but the ensemble model has higher recall values across all trajectory stretches and the independent set. This suggests that the ensemble model can better identify positive instances while maintaining a good level of precision. For the F1-score, which considers both precision and recall, it also shows improvement with the ensemble model compared to random forest, indicating better overall performance in terms of correctly identifying positive instances while minimizing false positives and false negatives.

The ROC-AUC scores are consistently higher for the ensemble model, indicating a better discrimination ability and a higher true positive rate across different thresholds compared to the random forest model. Both models show a trend of increasing performance metrics (accuracy, precision, recall, F1-score, and ROC AUC) as the trajectory stretch progresses from 0–20 ns to 80–100 ns. Performance on the independent set generally aligns with performance on the trajectory stretches, with both models showing similar trends in improvement. However, it is worth noting that the ensemble model consistently outperforms the random forest model on the independent set across all metrics.

In summary, the ensemble model shows significant improvement over the random forest model across all performance metrics, indicating its effectiveness in prediction tasks, especially in distinguishing native vs. non-native PPIs. Ensemble classifiers outperform individual models, in this case random forest, due to their ability to combine the unique strengths of diverse base learners through a meta-classifier, which strategically optimizes the ensembles’ overall predictions. This approach not only reduces the likelihood of over-fitting by averaging out individual model errors and biases but also enhances generalization to new data by leveraging different models’ sensitivities to various features of the dataset. In molecular dynamics simulations for protein–protein interactions, the varied and complex data benefit from an ensemble’s robustness to variability and its capacity to capture complex non-linear relationships, leading to improved performance metrics across all trajectory intervals. The logistic regression meta-classifier in the ensemble further fine-tunes predictions, resulting in a more accurate and reliable ensemble that adapts better to unseen data, as evidenced by the consistently higher accuracy, precision, recall, F1-score, and ROC AUC values.

## 3. Materials and Methods

### 3.1. Dataset and Feature Representation

**Figure 4:**
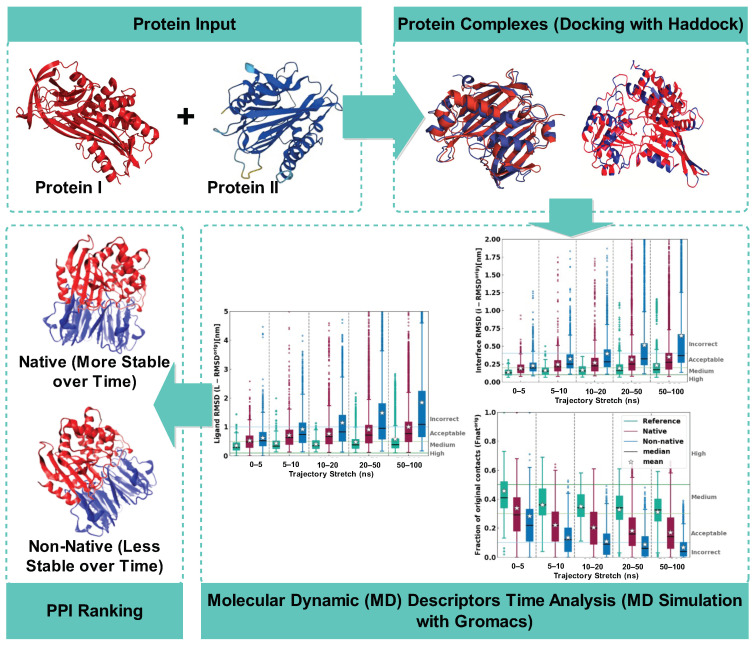
Distinguishing native from non-native protein–protein interactions: two different input proteins (distinguished by two different colors proteins) are first processed through protein docking using HADDOCK, generating potential protein complex models (illustrate as overlapping content). These complexes are then subjected to molecular dynamics (MD) simulations with GROMACS. The resulting MD trajectory data are used to rank the poses, identifying native and non-native PPIs.

Figure 4 illustrates a comprehensive data-driven approach for identifying biologically relevant protein–protein interactions. The process begins with two individual protein structures, Protein I and Protein II, as inputs. These proteins are computationally modeled to form a complex. In this study, 2030 complexes were selected from Docking Benchmark Version 5 and docked using HADDOCK version 2.4. Out of these, 25 complexes were chosen for MD simulation, which featured as a top-ranked cluster. Study defined two sets for training and validation, 20 common complexes as training and testing sets. The independent test set consisted of five complexes. Subsequently, these docked protein complexes underwent molecular dynamics (MD) simulations employing GROMACS, a powerful tool that allows for the observation of protein behavior over time under simulated physiological conditions. Key stability indicators such as root mean square deviations and fraction of native contacts were extracted throughout the MD simulation to analyze the time-dependent stability of each complex. Finally, the complexes were ranked based on their stability profiles, effectively distinguishing between native complexes, which display higher stability and retain their structural integrity over time, and non-native complexes, which demonstrate less stability and greater deviations from their initial conformation. This sophisticated method enabled researchers to efficiently screen for and validate potential protein interactions, paving the way for deeper insights into biological functions and mechanisms.

In this paper, two distinct training and independent datasets, both obtained from study [70], were utilized to develop a prediction model for PPI sites and assess its performance in comparison to other established prediction methods. This investigation utilized a rigorously balanced dataset, partitioned into five cohorts based on trajectory intervals, with each cohort containing 6720 entries of native protein–protein interactions (PPIs) and an equivalent number of non-native PPI entries. Of the total dataset, 80% was allocated for training purposes, while the remaining 20% was designated for testing. Additionally, for the final validation phase, the independent dataset was similarly divided into five discrete files according to trajectory intervals, with each file comprising 1680 entries of native PPIs and 1680 entries of non-native PPIs. A detailed description of all the datasets used in this research is provided in Figure S7 of the Supplementary Document. An additional analysis was conducted on 25 complexes using molecular dynamics (MD) simulations. Five complexes were designated as an independent test set to evaluate the model. The dataset comes with eight independent variables, denoted as *x* in Equations (1), (2), (9)–(14); they are RMSd_l, RMSd_i, dFnat, dBSA, dNonb_e, dNonb_water, dcom_distance, and dhbnum. Each of the eight features provided from MD simulations plays a specific role in differentiating between native and non-native protein–protein interactions (PPIs). The term “native” refers to interactions that occur under physiological conditions within an organism; on the other hand, “non-native” refers to interactions that occur under non-physiological conditions [71,72]. Native interactions, on average more stable [73], involve properly folded proteins in their native conformations, engaging in specific and biologically relevant interactions that contribute to various cellular processes [74], while non-native interactions involve proteins that are not properly folded, leading to aberrant protein conformations [75]. Non-native interactions may not contribute to normal cellular functions and can sometimes lead to aggregation, dysfunction, or even disease states [76,77]. Therefore, the distinction between native and non-native interactions is crucial in understanding the physiological relevance and implications of protein interactions within biological systems.

Ligand Root Mean Square Deviation (RMSd_l) measures the deviation of the ligand protein’s position compared to a reference structure after superimposing the backbone atoms, and indicates how closely the predicted interaction matches the native state [78,79,80]. Lower RMSd_l values indicating that the ligand’s position closely matches the position in the native complex, while higher RMSd_l values are expected as non-native interactions can result in significant deviations from the native ligand position due to improper folding [81,82]. The Interface Root Mean Square Deviation, RMSd_i, measurement is focused on the interface of the interacting proteins. High RMSd_i values result from significant alterations in the structure of one monomer when it binds [83]. The fraction of common contacts (Fnat) represents the proportion of native interfacial interactions that remain in the predicted docked complex’s interface in comparison to the experimental complex structure [84]. A higher Fnat value indicates that a large proportion of native contacts is maintained, while a lower Fnat value signifies the loss of native contacts, which might lead to loss of function or altered biological activity [41]. The buried surface area (BSA) quantifies the extent of the interface in a protein–protein complex [85]. Delta Non-bonded Energy (dNonb_e) refers to changes in non-bonded energy contributions. The non-bonded energy changes should be favorable, showing that the interaction is energetically stable and likely to occur in a physiological setting. Delta Non-bonded Water (dNonb_water) likely measures changes in non-bonded interactions involving water molecules, serving various roles in protein–protein complexes, such as facilitating hydrogen bonds between interacting partners due to their ability to function as both donors and acceptors [86]. The number of hydrogen bonds (dhbnum) counts the number of hydrogen bonds formed in the complex. A detailed explanation of how MD simulations can differentiate native and non-native PPIs can be seen in the Supplementary Documents.

### 3.2. Baseline Classifier

Breimen created the idea of random forests in 2001, which are made up of an array of tree prediction algorithms in which the performance of each tree is determined by the values of a random vector that is sampled independently and distributes identically across all trees in the forest [87]. The idea in question was initially introduced by Ho in 1995 [88] when he introduced the random decision forest concept. An infinite number of decision trees may be appended and it addresses the issue wherein a solitary decision tree is susceptible to over-fitting. A pair methods for randomization are utilized: first, data is sampled at random for bootstrap samples, and subsequently, input attributes are selected at random for the construction of individual base decision trees [89]. Random forest will train *M* decision trees on bootstrap samples; however, during tree construction, only a random subset of all predictors is considered at each split [90]. The process for creating a random forest, comprising *N* trees, is outlined as follows [91]:

1. Generate a bootstrap sample, denoted as $X_{n}$, for each tree.
2. Develop each tree, labeled as $T_{n}$, utilizing the respective sample $X_{n}$.
3. Determine the optimal predictor for each split of the tree by selecting from random subsets of predictors, guided by a predefined criterion such as entropy or the Gini index.

The gradient boosting, first introduced by Friedman et al. [92], aggregates the outcomes of multiple fundamental predictors in order to generate a robust ensemble that outperforms the combined performance of its individual parts [93]. The following describes the function estimation for the gradient boosting algorithm [94]:

1. We consider that we have a dataset with an input variable of $x=(x_{1},x_{1},...,x_{d})$ and correspondence labels of *y*, so that the dataset can be written as
  (9) $${\left(x,y\right)}_{i=1}^{N}.$$
2. The purpose is to estimate the function $\hat{f}\left(x\right)$ using a minimal loss function ψ(y,f) while reconstructing the unknown functional dependence of *x* on *y*, $x\overset{f}{\to }y$.
  (10) $$y=\hat{f}\left(x\right)=\underset{f(x)}{arg\min}\;\psi \left(y,f\left(x\right)\right)$$
3. The estimation equation can be reformulated by minimizing the expected loss function throughout the response data $E_{y}\left(\phi \left[y,f\left(x\right)\right]\right)$:
  (11) $$y=\hat{f}\left(x\right)=\underset{f(x)}{arg\min}\;E_{x}\left[E_{y}\left(\phi \left[y,f\left(x\right)\right]\right)\;|\;x\right]$$

Extreme gradient boosting (XGBoost) is a extensible machine learning system for tree boosting [95], designed to be exceptionally lightweight, adaptable, and powerful [96]. XGBoost is a parallel tree-boosting technique that solves a wide variety of data science issues with lightning-fast performance and accuracy [97]. The XGBoost algorithm operates as follows:

1. We consider that we have a dataset with an input variable of $x=(x_{1},x_{1},...,x_{d})$ and correspondence labels of *y*, so that the dataset can be written as
  (12) $${\left(x,y\right)}_{i=1}^{N}$$
2. When *K* trees comprise an XGBoost, the resulting model is denoted as $\sum_{k=1}^{K}f_{k}$, where $f_{k}$ represents the prediction function of *k*th tree.
3. The following stage is calculating the expected output $\hat{y}$:
  (13) $${\hat{y}}_{i}=\sum_{k=1}^{K}f_{k}\left(x_{i}\right)$$
  where $x_{i}$ denotes the feature vector associated with the *i*th data point.

In order to mitigate the drawbacks associated with gradient boosting models [98] that perform unsatisfactorily when the feature dimension is high and the data size is large, Guolin Ke et al. introduced LightGBM, a technique consisting of Gradient-based One-Side Sampling (GOSS) and Exclusive Feature Bundling (EFB) [99]. The LightGBM algorithm is capable of aggregating distinct attributes into a consolidated features, which may be utilized to construct histograms that categorize similar attributes [100]. The objective of the LightGBM algorithm, when applied to a supervised dataset *X*, is to identify an estimation of the function $\hat{f}\left(x\right)$ that reduces the expected value of a chosen loss function L(y,f(x)) [101]:

(14) $$\hat{f}\left(x\right)=argmin_{f}\;E_{y,X}\;L\left(y,f\left(x\right)\right)$$

## 4. Conclusions

The results of our investigation into the predictive capabilities of ensemble classifiers for protein–protein interactions (PPIs) reveal a substantial advancement over traditional single-algorithm approaches, particularly in the nuanced differentiation between native and non-native interactions. The methodology combines the strengths of multiple machine learning models through a logistic regression meta-classifier. Our results showed that while improvements in AUC and other metrics might seem modest, they contribute to a more robust, consistent, and adaptable model. To evaluate the effectiveness of four baseline models, we compared their performance to logistic regression. This comparison highlighted that logistic regression consistently underperformed across all metrics, suggesting that it is not powerful enough to capture the complex interactions within the data. This makes tree-based models a more suitable choice for simulating molecular dynamics.This research underscores the critical importance of adopting multifaceted, integrative strategies to address the complexity inherent in biological systems, especially in the context of PPIs, which are pivotal to understanding cellular function and disease mechanisms. By providing a more accurate and robust tool for identifying potential PPI sites, we pave the way for the development of novel therapeutic strategies that target specific protein interactions, potentially revolutionizing the approach to treating a myriad of diseases. Furthermore, this study contributes to the foundational knowledge necessary to navigate intricate biological pathways. In conclusion, the success of our ensemble classifier in predicting PPIs not only marks a significant milestone in computational biology but also serves as a compelling testament to the power of machine learning in enhancing our understanding of biological complexity. 

## Figures and Tables

**Table 1:** Model performance of previous and proposed model on each trajectory stretch for testing and independent set.

Model	Evaluation	Testing Data, at Each Trajectory Interval					Independent Set
		**0–20 ns**	**20–40 ns**	**40–60 ns**	**60 –80 ns**	**80–100 ns**	
Previous Model [70]	Accuracy	0.77	0.83	0.85	0.85	0.86	0.60
	Precision	0.79	0.86	0.87	0.86	0.88	0.61
	Recall	0.76	0.81	0.84	0.84	0.85	0.61
	F1-Score	0.76	0.82	0.85	0.84	0.85	0.59
	ROC AUC	0.86	0.92	0.93	0.93	0.94	0.60
Ours	Accuracy	0.84	0.89	0.91	0.92	0.92	0.63
	Precision	0.84	0.90	0.91	0.93	0.92	0.61
	Recall	0.83	0.89	0.91	0.92	0.92	0.74
	F1-Score	0.84	0.89	0.91	0.92	0.92	0.63
	ROC AUC	0.92	0.96	0.97	0.98	0.97	0.63

## Data Availability

The datasets and code used in this study are available for download at https://github.com/caecarnkcp/PPI, accessed on 28 May 2024. These resources are publicly accessible without any restrictions, ensuring transparency and facilitating the reproducibility of the study’s results.

## Supplementary Materials

The following supporting information can be downloaded at https://www.mdpi.com/article/10.3390/ijms25115957/s1.

## Abbreviations

The following abbreviations are used in this manuscript:

AUC	Area Under the Curve
CNN	Convolutional Neural Network
dBSA	Delta Buried Surface Area
dCom_*Distance*_	Delta Center of Mass Distance
dFNat	Delta Fraction Native
dHBNum	Delta Hydrogen Bond Number
DNN	Deep Neural Network
dNonb_*E*_	Delta Non-bonded Energy
dNonb_*Water*_	Delta Non-bonded Water
EFB	Exclusive Feature Bundling
EMs	Electron Microscopy
FNs	False negatives
FPs	False positives
GANs	Graph attention networks
GCNs	Graph convolutional networks
GE	Global Encoding
GOSS	Gradient-based One-Side Sampling
LGB	Light gradient boosting
MCC	Matthew’s correlation coefficient
MD	Molecular dynamics
NMR	Nuclear Magnetic Resonance
QSAR	Quantitative Structure–Activity Relationships
RMSd-i	Root Mean Square Deviation of Interface
RMSd-l	Root Mean Square Deviation of Ligand
ROC	Receiver Operating Characteristic
TNs	True negatives
TPs	True positives
XGBoost	Extreme gradient boosting
LR	Logistic regression
NB	Naïve Bayes
NN	Neural Network
PPIs	Protein–protein interactions
RF	Random forest
SVMs	Support Vector Machines
