# Supplementary material for: An Ensemble Classifiers for Improved Prediction of Native–Non-Native Protein–Protein Interaction
Source: Int J Mol Sci. 2024 May 29;25(11):5957. doi: 10.3390/ijms25115957 (PMC11172808; doi:10.3390/ijms25115957)
Supplement: Supplementary file 1 [file ijms-25-05957-s001.zip › ijms-3000160-supplementary.pdf]

Supplementary Document

1. Ensemble Classifier for Native-Non Native PPI Algorithm

Algorithm S1 Ensemble Classifier for Native-Non Native PPI

Input: Training set $D = \{x_i, y_i\}_{i=1}^{k} \rightarrow (x_i \in \Re, y_i \in \Re)$, y_i is set of possible class (1 for native, and 0 for non-native)
Base models: First level baseline classifiers $h_1, h_2, ..., h_j$.
Evaluation criteria: Acc, Pre, Rec, F1-Score, MCC

Step 1: Develop the first-level baseline model classifier
for $j \leftarrow 1$ to J **do**
  Develop a baseline classifier h_j within the data D
end for
Step 2: Create the new dataset from D
for $i \leftarrow 1$ to k **do**
  New Dataset $\hat{D} = \{\hat{x}_i, y_i\}, \hat{x}_i = \{h_1(x_i), h_2(x_i), ..., h_j(x_i)\}$
end for
Step 3: Develop the second-level meta-model classifier
Stacking classifier $\hat{h}$ by utilizing the most recent dataset $\hat{D}$.
return $H(x) = \hat{h}\,(h_1(x), h_2(x), h_3(x), ..., h_j(x))$

2. The TP, TN, FN and FP Values for Each Model

Table **S1**: Confusion matrix showing the performance of a classification model differentiating between Native and Non-Native classes.

		Prediction Outcome	
		**Native (0)**	**Non-Native (1)**
Actual Value	**Native (0)**	True Positive (TP)	False Negative (FN)
	**Non-Native (1)**	False Positive (FP)	True Negative(TN)

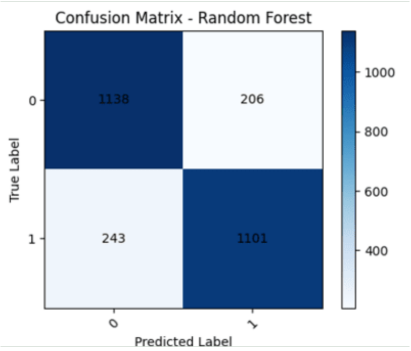

(a) Random Forest

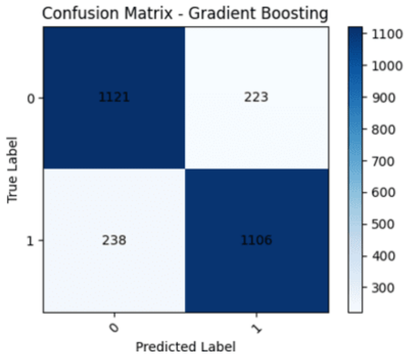

(b) Gradient Boosting

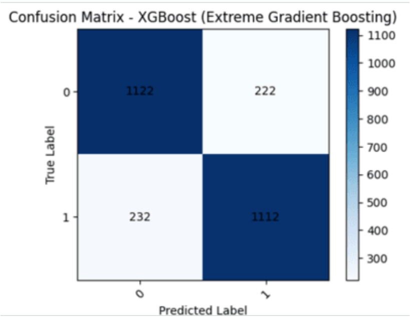

(c) Extreme Gradient Boost.

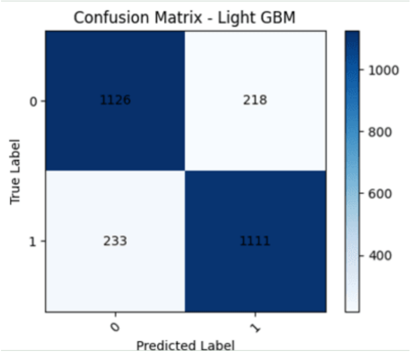

(d) Light Gradient Boost.

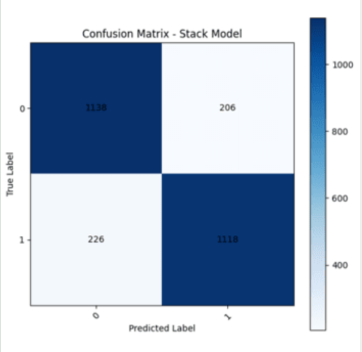

(e) Stacking Model

Figure **S1**: Confusion Matrix for 0-20 ns Trajectory Intervals, on Testing Dataset

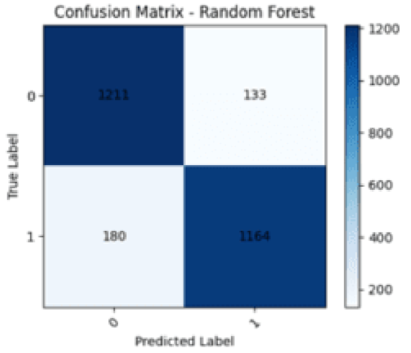

(a) Random Forest

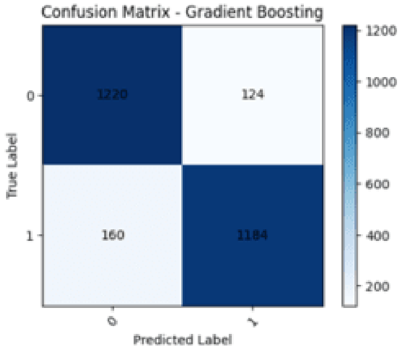

(b) Gradient Boosting

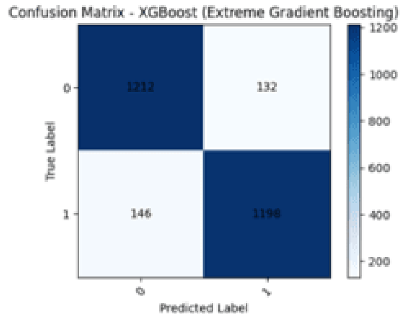

(c) Extreme Gradient Boost.

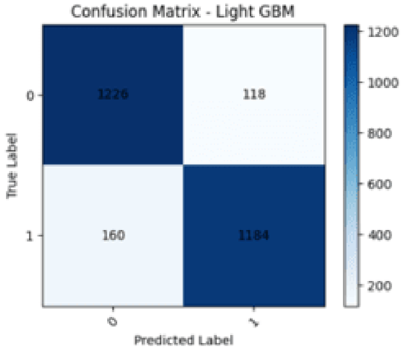

(d) Light Gradient Boost.

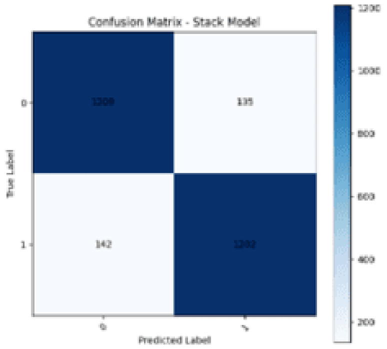

(e) Stacking Model

Figure **S2**: Confusion matrix for 20-40 ns Trajectory Intervals, on Testing Dataset

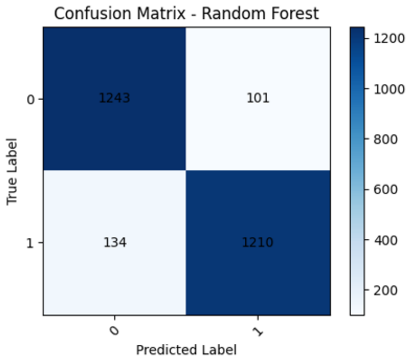

(a) Random Forest

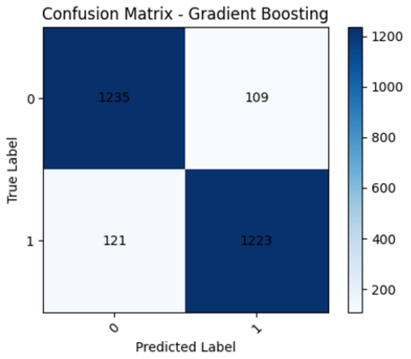

(b) Gradient Boosting

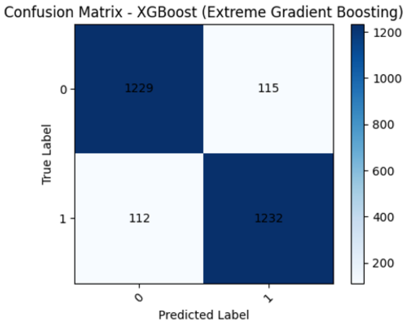

(c) Extreme Gradient Boost.

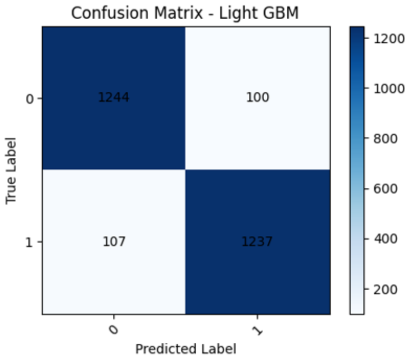

(d) Light Gradient Boost.

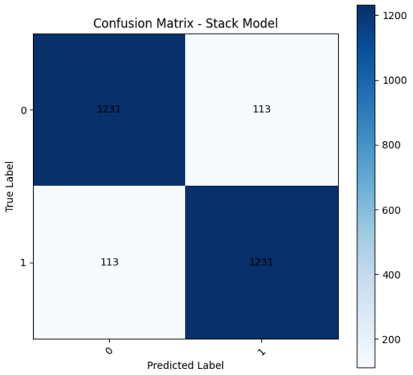

(e) Stacking Model

Figure **S3**: Confusion matrix for 40-60 ns Trajectory Intervals, on Testing Dataset

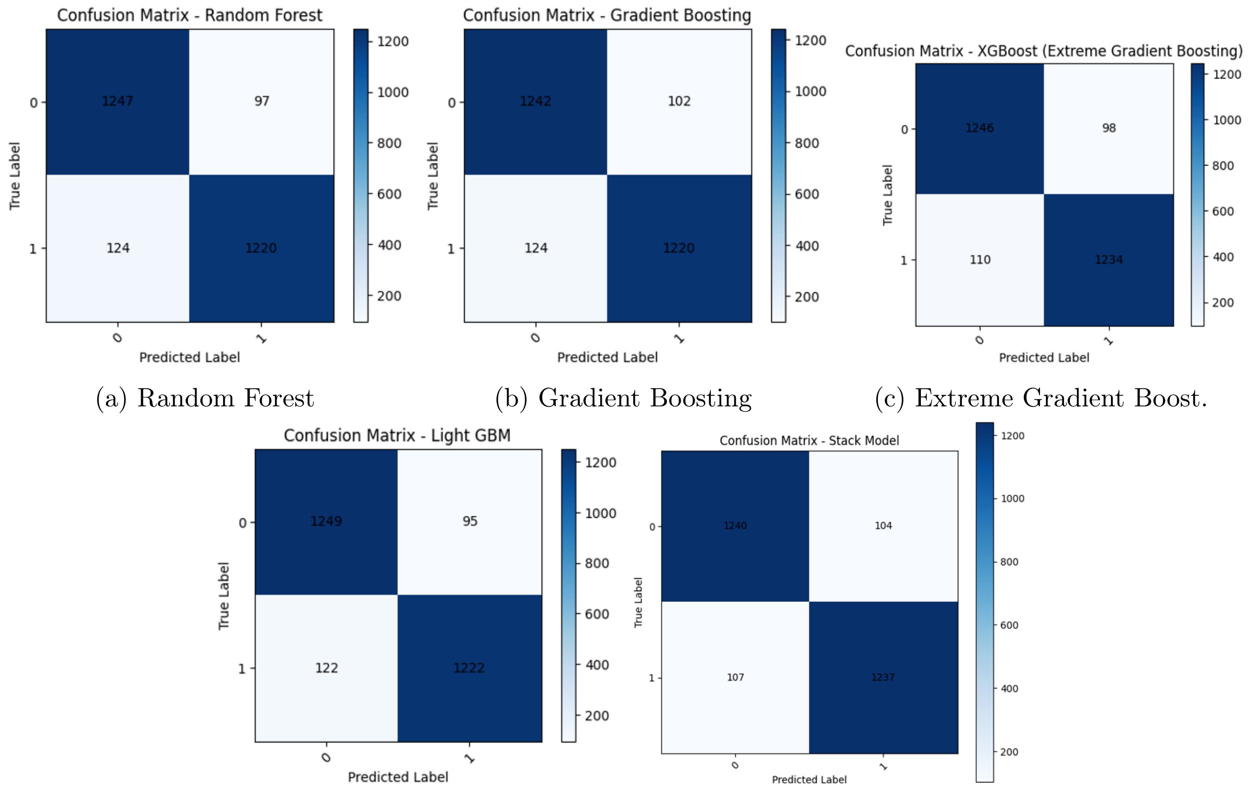

(a) Random Forest (b) Gradient Boosting (c) Extreme Gradient Boost.

(d) Light Gradient Boost. (e) Stacking Model

Figure **S4**: Confusion matrix for 60-80 ns Trajectory Intervals, on Testing Dataset

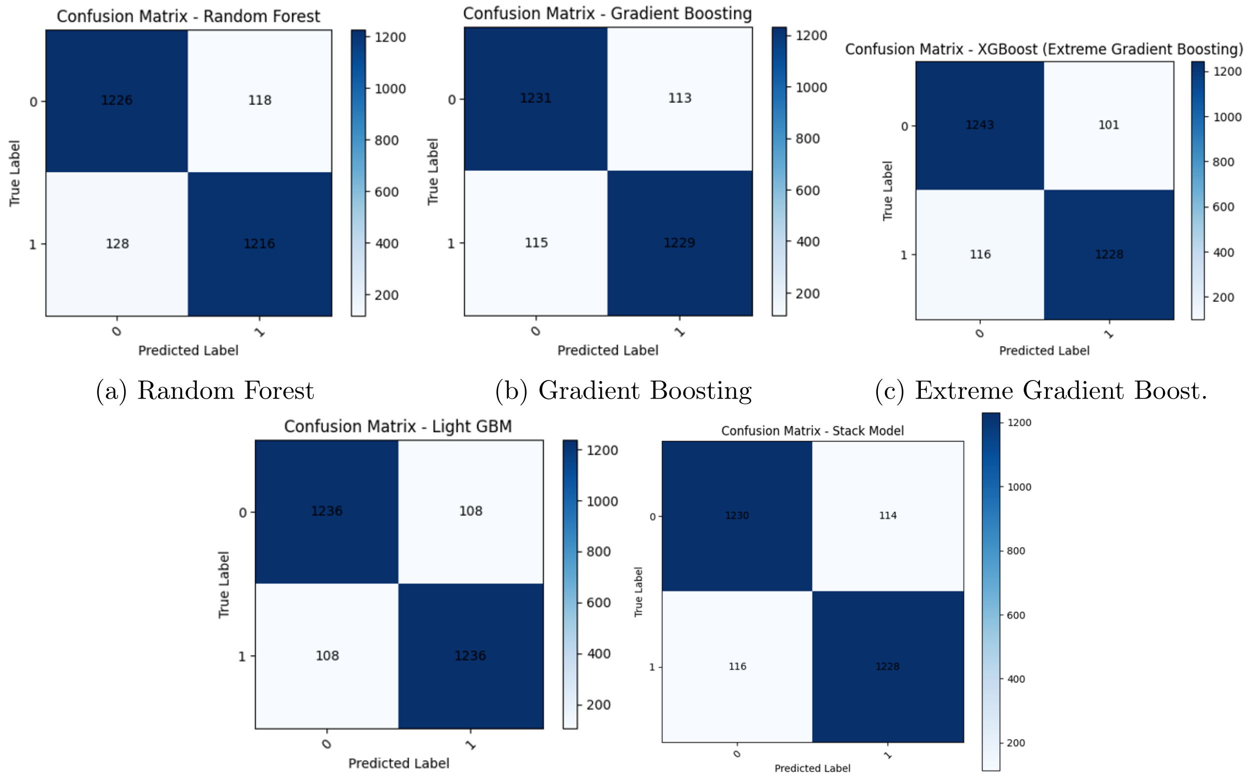

(a) Random Forest (b) Gradient Boosting (c) Extreme Gradient Boost.

(d) Light Gradient Boost. (e) Stacking Model

Figure **S5**: Confusion matrix for 80-100 ns Trajectory Intervals, on Testing Dataset

3. **Performance Metrics of Logistic Regression over Different Trajectory Intervals**

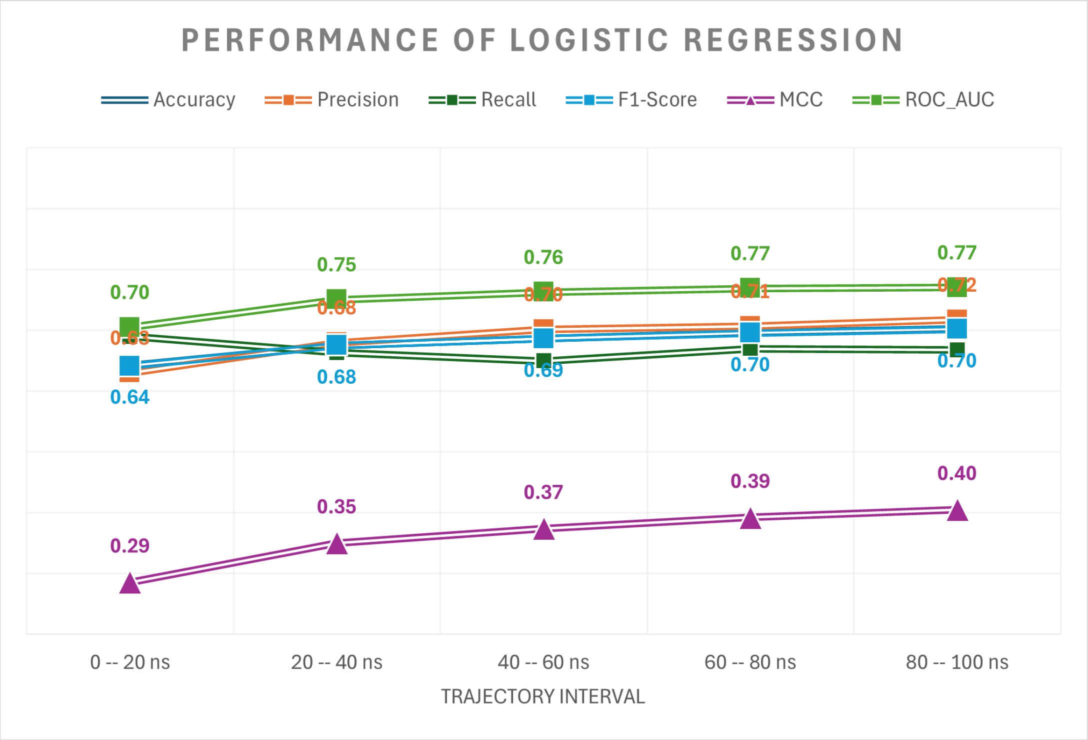

Figure **S6**: Performance metrics of logistic regression over different trajectory intervals. The metrics include Accuracy, Precision, Recall, F1-Score, MCC, and ROC_AUC.

4. **Training-Testing Dataset and Validation Dataset**
   This study utilized a rigorously balanced dataset, partitioned into five cohorts based on trajectory intervals, with each cohort containing 6720 entries of native protein-protein interactions (PPI) and an equivalent number of non-native PPI entries. For further reference and use, We have provided the dataset in the supplementary files under the name as mentioned below.

   (a) Training-Testing Dataset
      i. features_1_20.csv
      ii. features_20_40.csv
      iii. features_40_60.csv
      iv. features_60_80.csv
      v. features_80_100.csv

   (b) Validation Dataset (Independent Dataset)
      i. features_1_100.csv
      ii. features_1_20.csv
      iii. features_20_40.csv
      iv. features_40_60.csv
      v. features_60_80.csv
      vi. features_80_100.csv

   The datasets are available for download at the following link: https://github.com/caecarnkcp/PPI. These CSV files contain all the necessary data to reproduce our results and are accessible to the public without any restrictions.

   To address your concerns, we have provided a dataset tree diagram illustration that clarifies the structure and distribution of our data. The dataset tree diagram illustrates the distribution for each trajectory intervals.

   - **Training and Testing Phase**

  - For each trajectory interval (0-20 ns, 20-40 ns, 40-60 ns, 60-80 ns, 80-100 ns), the dataset consisted of 6720 native and 6720 non-native entries, totaling 13440 entries.
  - This dataset was split into 80% training data (10752 entries) and 20% testing data (2688 entries). Each subset was further balanced to contain equal numbers of native and non-native entries:
    * Training data: 5376 native and 5376 non-native entries.
    * Testing data: 1344 native and 1344 non-native entries.

  The random selection process ensures that both the training and testing sets are representative samples of the entire dataset, maintaining the balance between native and non-native entries. This method enhances the robustness and generalizability of the model by preventing any biases in the data splitting.

- **Validation Phase**
  - The independent validation dataset, used to test the model's generalizability, included 1680 native and 1680 non-native entries, totaling 3360 entries for each trajectory interval.

  This independent dataset was kept separate from the training and testing sets and was used exclusively for final model validation. For the validation process, the model was simulated for the full 100 ns without breaking it into smaller intervals. This approach aimed to assess the model's performance over an extended period, providing a more comprehensive evaluation of its ability to distinguish between native and non-native protein-protein

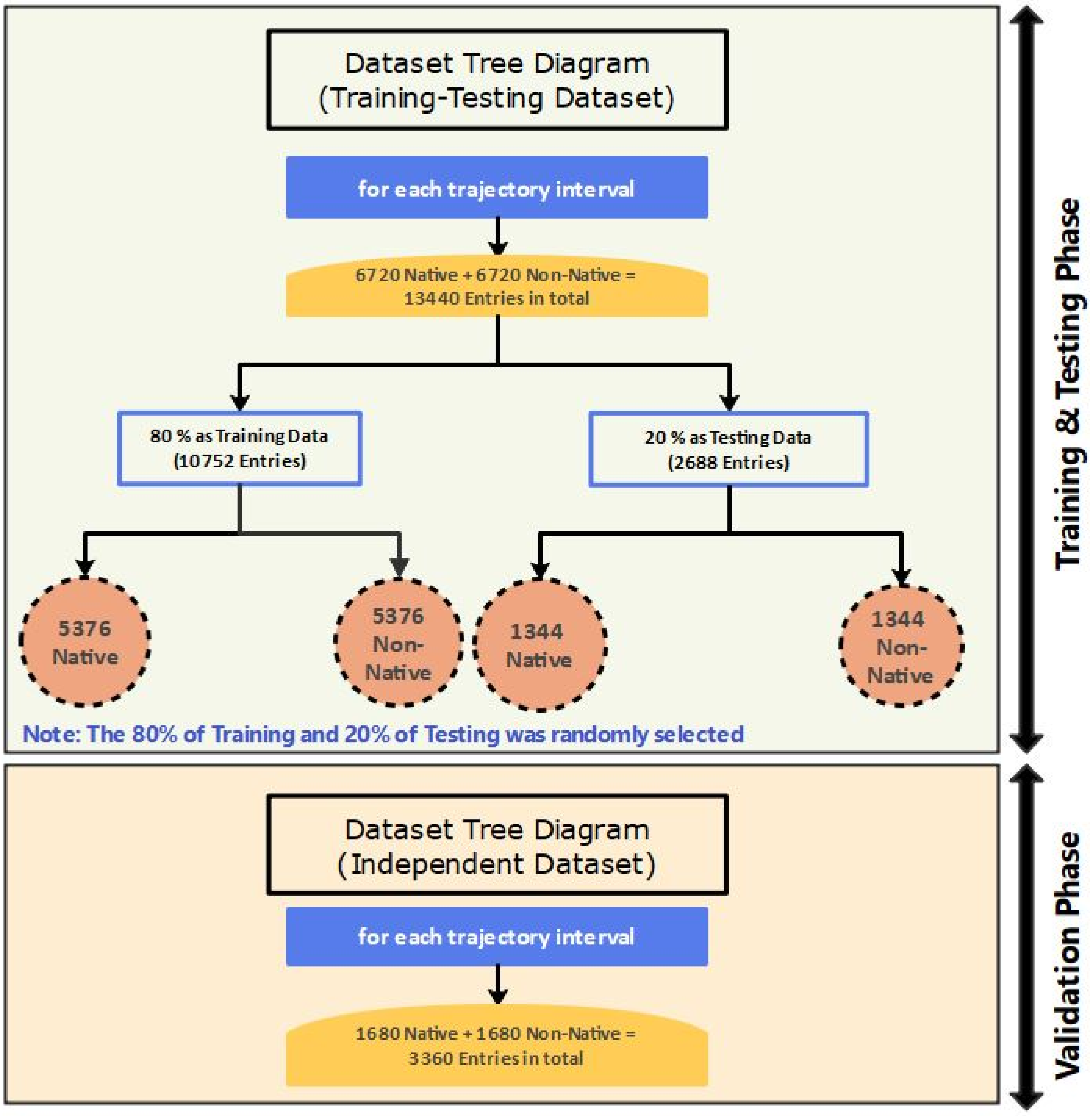

Figure **S7**: Dataset Tree Diagram

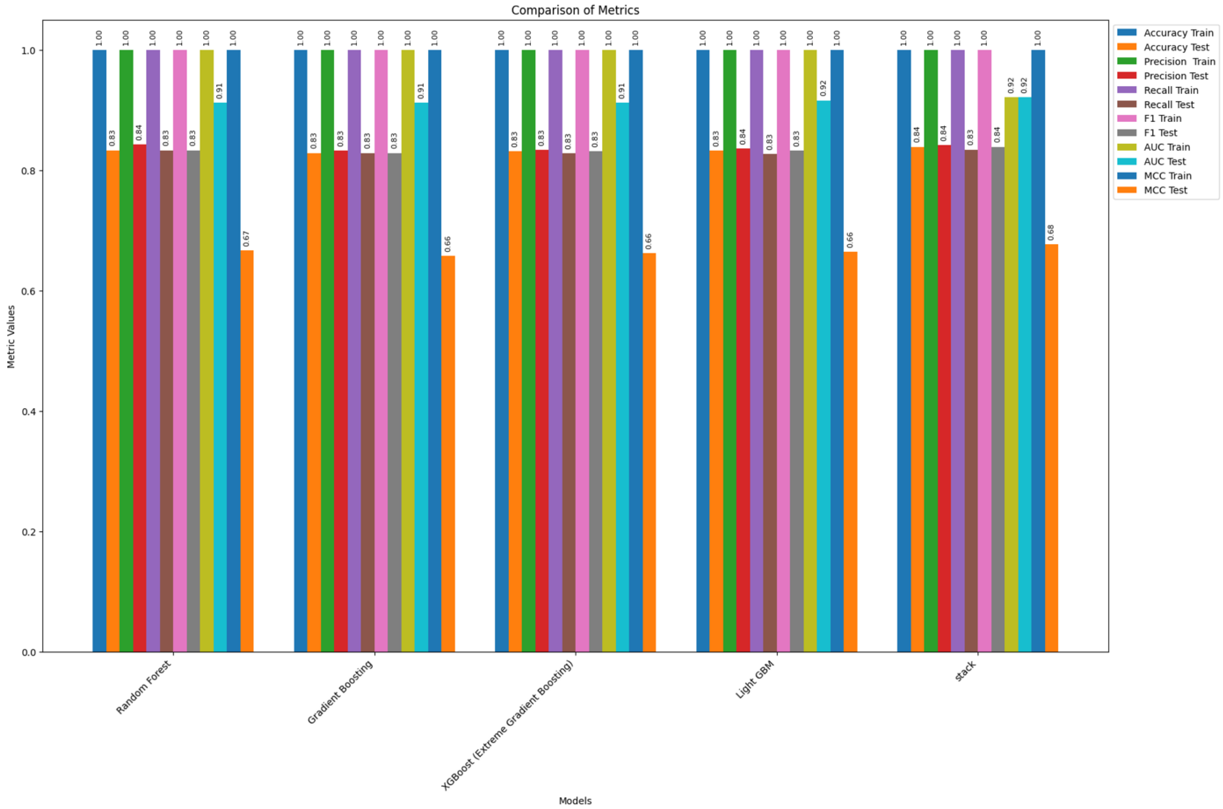

Figure **S8**: Performance comparison of different machine learning models for predicting Native vs. Non-Native PPIs, at 0–20 ns trajectory interval. The metrics displayed for both training and testing datasets

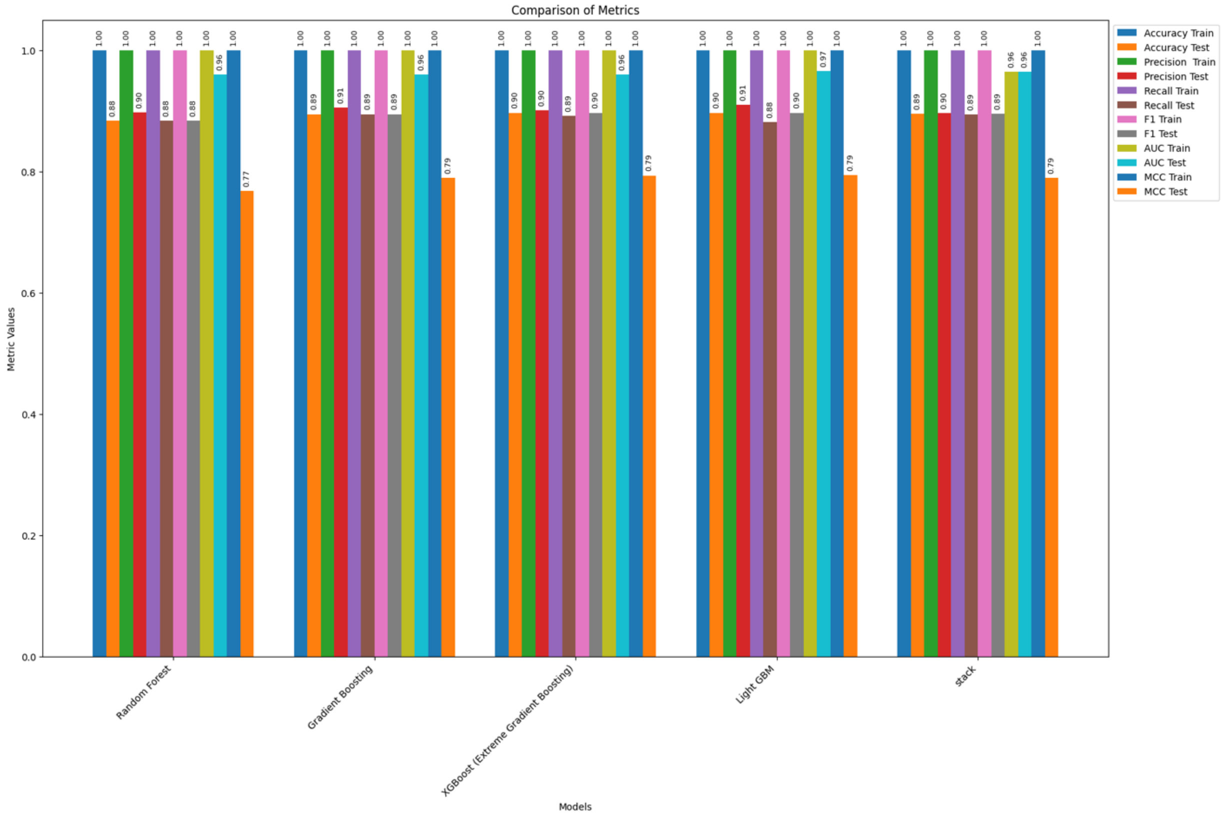

Figure **S9**: Performance comparison of different machine learning models for predicting Native vs. Non-Native PPIs, at 20–40 ns trajectory interval. The metrics displayed for both training and testing datasets

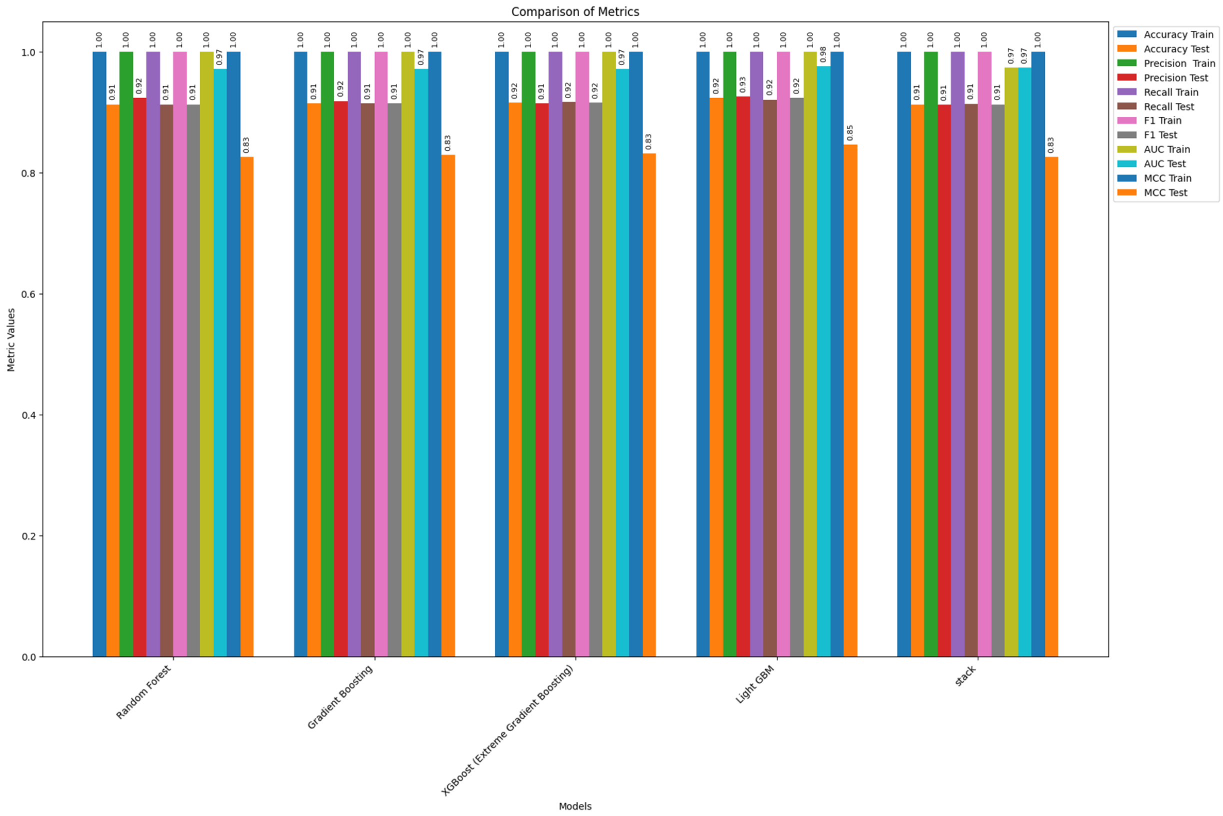

Figure **S10**: Performance comparison of different machine learning models for predicting Native vs. Non-Native PPIs, at 40–60 ns trajectory interval. The metrics displayed for both training and testing datasets

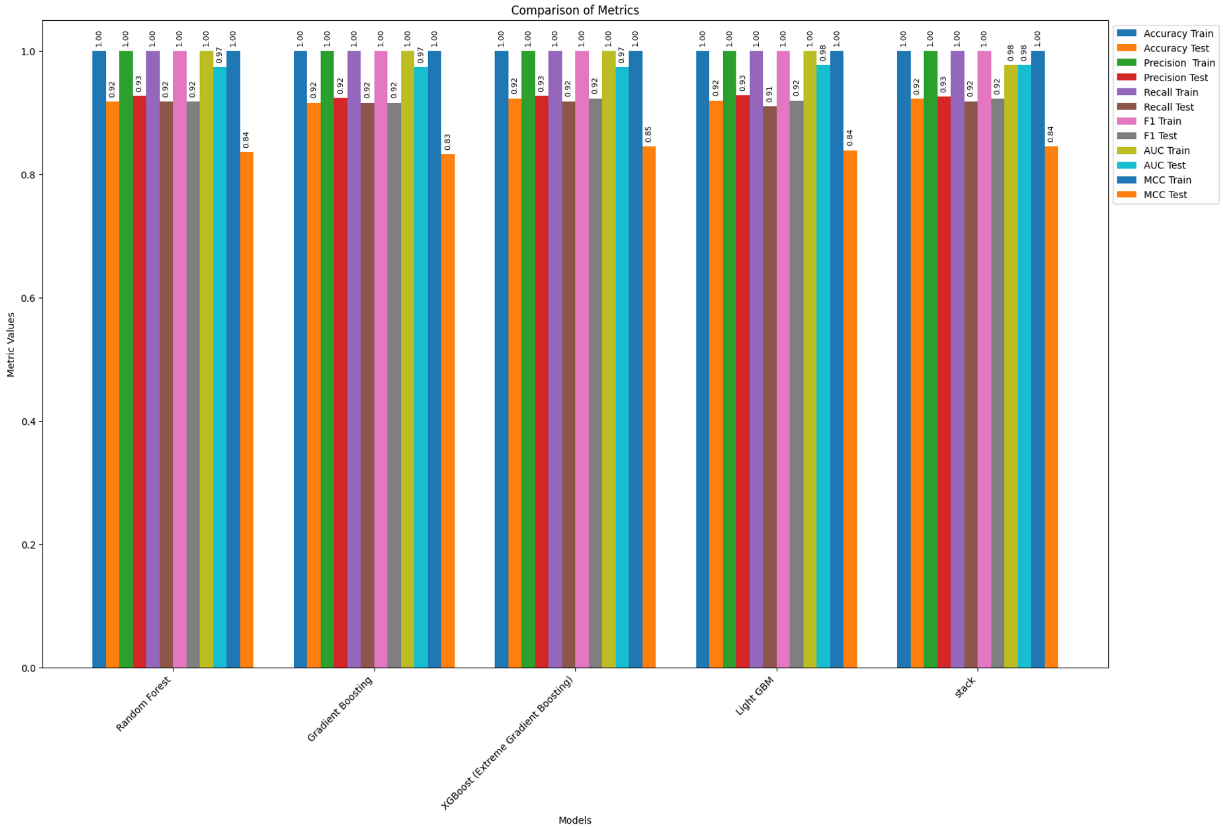

Figure **S11**: Performance comparison of different machine learning models for predicting Native vs. Non-Native PPIs, at 60–80 ns trajectory interval. The metrics displayed for both training and testing datasets

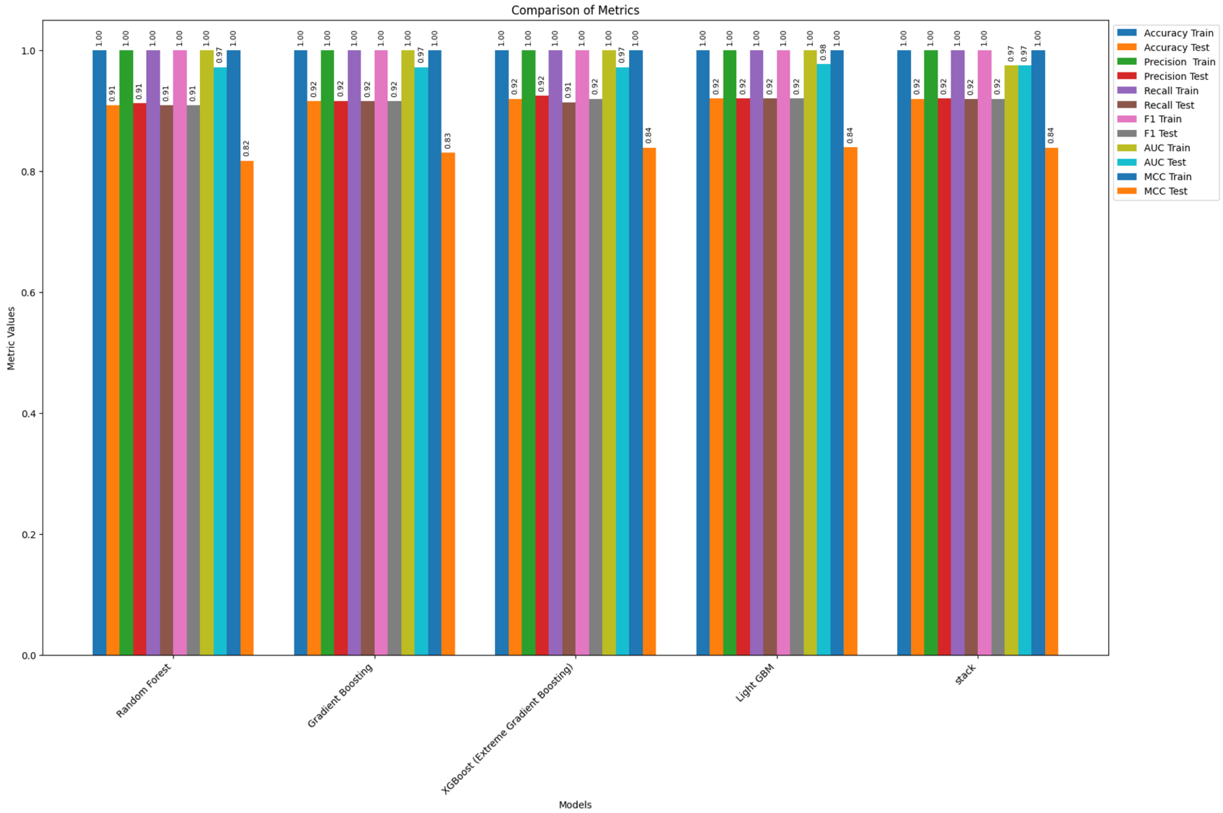

Figure **S12**: Performance comparison of different machine learning models for predicting Native vs. Non-Native PPIs, at 80–100 ns trajectory interval. The metrics displayed for both training and testing datasets

The training data is used to train machine learning models, allowing the algorithms to learn patterns or features in the data, optimize internal parameters, and fit the model by identifying relationships between inputs and outputs. Testing data evaluates the model's performance post-training, measuring metrics such as accuracy, precision, recall, F1 score, and AUC to understand the model's strengths and weaknesses, ensuring it generalizes well to unseen data and isn't over-fitting. The validation data (independent dataset) provides an objective assessment of the model's performance, separate from the training and testing data, used for measuring generalization capability, and preventing evaluation bias. Figure **S8**, **S9**,**S10**,**S11**,and **S12** provided the Training and Testing performance for each model.

5. **How MD Simulations Distinguish between Native and Non-Native Complexes based on CAPRI Measures?**
Molecular dynamics (MD) simulations can effectively distinguish between native and non-native protein complexes. They do this by observing how stable the complexes are, how well they retain contact points, and how they change structurally over time. These differences can be measured using the Critical Assessment of Prediction of Interactions (CAPRI) standards.The MD can distinguish between the behaviors of native and non-native models, although in practical situations, a reference structure is often unavailable. To evaluate this, prior study [1] chose the model at the beginning of each production run to serve as **a reference (ground truth)**. Prior study provide the characteristics Fnat, l-RMSD and i-RMSD which has been compared with the reference point, then this there parameter called as $Fnat^{orig}, l-RMSD^{orig}$ and $i-RMSD^{orig}$. This parameter highlight their behavior relative to the initial binding mode (reference/ground truth). The simulations track how much the simulated complexes deviate from this initial model. Reference structures generally show the lowest deviations, indicating they are more stable than the simulated models. Both ligand and interface $RMSD^{orig}$, the reference structures show the lowest values, pointing to the (expected) higher stability of the experimental complexes compared to their docked models. More interestingly, the near-native complexes show overall higher stability (less deviations from the initial values) than the non-native ones. Even though the distributions of both $RMSD^{orig}$ are largely overlapping, their means are clearly distinguishable at the end of the simulation. While for the fraction of native contacts $Fnat^{orig}$ is depicted for native, non-native, and reference structures over different trajectory time stretches. The reference contacts show a generally stable and high fraction across all time intervals. While, the native contacts vary more widely than the reference contacts. The median values generally trend lower than the reference. However,

the native contacts start near reference levels in the earliest interval (0-5 ns). The non-native contacts consistently have the lowest fraction across all intervals. The medians are significantly lower than the reference, indicating a much less frequent maintenance of original structural contacts compared to the reference. The reference structures consistently maintain a high fraction of original contacts. Native structures, while closer to the reference than non-native ones, still exhibit a drop, particularly in longer time frames. Non-native structures consistently show the lowest retention of original contacts across all considered time frames.

References

[1] Zuzana Jandova, Attilio Vittorio Vargiu, and Alexandre MJJ Bonvin. "Native or Non-Native Protein–Protein Docking Models? Molecular Dynamics to the Rescue". In: *Journal of Chemical Theory and Computation* 17.9 (2021), pp. 5944–5954.
